# Effects of Cadmium Exposure on Age of Menarche and Menopause

**DOI:** 10.3390/toxics6010006

**Published:** 2017-12-27

**Authors:** Xiao Chen, Guoying Zhu, Taiyi Jin

**Affiliations:** 1 Department of Nephrology, Zhongshan Hospital, Fudan University, 180 Fenglin Road, Shanghai 200032, China; chxwin@163.com; 2 Institute of Radiation Medicine, Fudan University, 2094 Xietu Road, Shanghai 200032, China; zhugy@shmu.edu.cn; 3 Department of Occupational Medicine, School of Public Health, Fudan University, 150 Dongan Road, Shanghai 200032, China

**Keywords:** cadmium, female, menarche, menopause

## Abstract

Cadmium exposure can cause several adverse health effects. Animal studies have also shown that cadmium exposure can affect menarche or menopause. However, data is limited in humans. We conducted a retrospective study to assess whether cadmium exposure was associated with different ages of menarche and menopause in a Chinese population. A total of 429 women living in control (*n* = 137) and two cadmium-polluted areas (*n* = 292) were included in this study. A total of 223 and 206 subjects were included in the analysis for menarche and menopause, respectively. The median menarche age of population living in the heavily cadmium-polluted area was significantly younger than those in the control area (14.0 vs. 15.0, *p* < 0.01). Logistic regression showed that the odds ratio (OR) of early occurrence of menarche (<13 years) in the population living in the heavily polluted area and moderately polluted area was 3.7 (95% confidence interval (CI): 1.5–9.7) and 1.3 (95% CI: 0.7–2.6) compared with control, respectively. No significant difference was observed in the age of menopause in the population of these three areas. In conclusion, our data indicated that cadmium exposure may cause early menarche.

## 1. Introduction

Cadmium exposure has been shown to adversely affect the liver, kidney, cardiovascular system, and bones. Cadmium also acts as an endocrine disruptor and affects reproduction system [1]. Animal studies have shown that cadmium exposure can cause an increase of uterine wet weight, endometrial thickness, and endometrial stromal thickness in female rats [2]. Studies also indicated that maternal exposure to cadmium can increase early delivery and lower birth weight [3,4]. However, very little human data were available on the association between environmental level of cadmium exposure and age of menarche and menopause [5]. In the present study, we examined the association of cadmium exposure and menarche age and menopause age in a Chinese population.

## 2. Materials and Methods 

### 2.1. Study Area and Population

A ChinaCad study was performed during 1997, which aimed to observe the influence of cadmium exposure on renal dysfunction and osteoporosis [6]. The following three areas were included in the present study: Nanbaixiang (‘moderate’ near Wenzhou, cadmium in rice = 0.51 mg/kg), Jiaoweibao (‘heavy’ near Wenzhou, mean cadmium concentration in rice = 3.7 mg/kg), and a control area (40 km from Wenzhou, Cd concentration in rice = 0.07 mg/kg). There was a smelter in the heavily polluted area that began production in 1961. The waste water was directly discharged into the river. Residents living in the polluted areas used the polluted river water to irrigate their fields from 1961 to 1995. The cadmium concentration in rice was 3.7 mg/kg in 1997. An area with similar nutrition and socioeconomic factors and low cadmium exposure was selected as the control. All subjects gave their informed consent for inclusion before they participated in the study. The study was conducted in accordance with the Declaration of Helsinki, and the protocol was approved by the Ethics Committee of Fudan University. 

A total of 488 women were included in this study. As the pollution started from 1961, we analyzed the association between menarche age and cadmium exposure in subjects <47 years and the association between menopause age and cadmium exposure in people that are 50–80 years old. A total of 429 women was finally included in this study, including 137 women in control area and 292 women in two cadmium polluted areas. A total of 223 subjects was included in menarche study and 206 were included in the menopause study. The information on menarche age and menopause age were obtained through self-reporting by subjects. 

### 2.2. Statistical Analysis

The data was analyzed using SPSS 16.0 (SPSS Inc., Chicago, IL, USA). Quantitative data were shown as means ± standard deviation or standard error and were analyzed by one-way analysis of variance (ANOVA) or analysis of covariance (adjusted with exposure duration). An earlier age of menarche was defined as age of <13 years old, while delayed menopause was defined as menopause age of >51 years old. Logistic regression was used to show the risk of an abnormal menarche age at different levels of cadmium exposure. *p*-values of less than 0.05 were considered to be statistically significant.

## 3. Results

**Figure 1:**
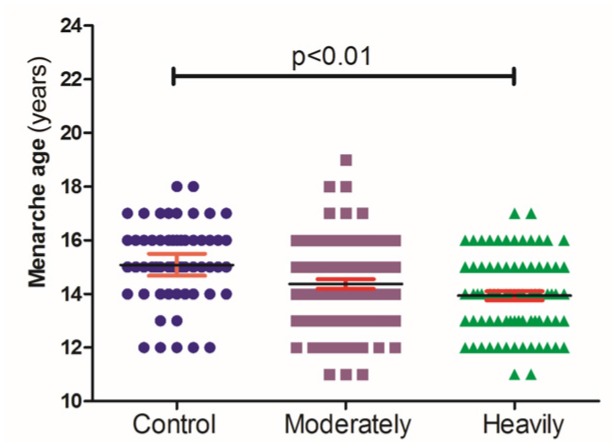
The menarche age of women in control (*n* = 57), moderately (*n* = 89) and heavily (*n* = 77) polluted areas.

**Figure 2:**
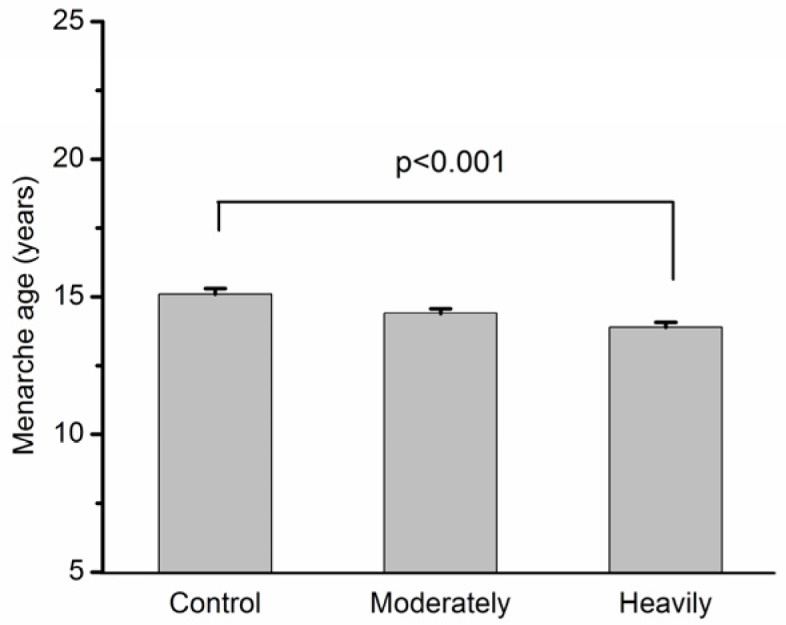
The menarche age of women in control (*n* = 57), moderately (*n* = 89) and heavily (*n* = 77) polluted areas after adjusting with exposure duration.

The median menarche age was 14 (11–16) in the heavily polluted area, 14 (11–18) in the moderately polluted area, and 15 (12–17) in the control area. The mean age of menarche was 13.9 in heavily polluted area, 14.4 in moderately polluted area, and 15.2 in control area (Figure 1), respectively. After adjusting for the exposure duration, the mean age of menarche was 14.0 in the heavily polluted area, 14.4 in the moderately polluted area, and 15.0 in the control area (Figure 2). The menarche age was approximately one year younger for subjects in the heavily polluted area compared with the subjects in the control area. The menarche age of subjects living in the heavily polluted area was significantly younger than those in the control area (*p* < 0.001). An earlier onset of menarche was observed in 6.5%, 12.5%, and 21.1% of subjects living in the control, moderately polluted area, and heavily polluted area, respectively. 

**Figure 3:**
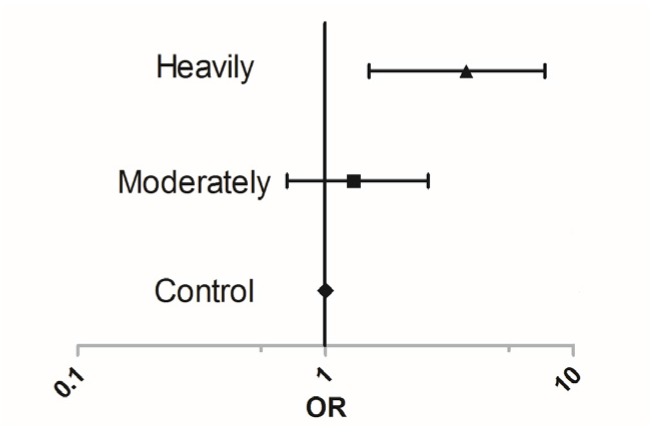
The odds ratio (OR) of early occurrence of menarche and cadmium exposure (control (*n* = 57), moderately (*n* = 89), and heavily (*n* = 77) polluted areas).

Logistic regression further showed that the odds ratio (OR) of early occurrence of menarche in the population in the heavily polluted area was 3.7 (95% confidence interval (CI): 1.5–9.7) compared with those people living in the control area (Figure 3). The population living in moderately polluted area also had a high risk of early menarche, but this was not statistically significant (OR = 1.3, 95% CI: 0.7–2.6). 

The menopausal age was 47.4, 47.5, and 47.0 in the women living in the heavily polluted area, moderately polluted area, and control area, respectively. After adjustment for exposure duration, the menopausal age was 47.3, 47.5, and 47.1, respectively. No significant differences were observed. A delayed menopause was observed in 12.3%, 15.1%, and 17.6% of subjects living in the control, moderately polluted, and heavily polluted areas, respectively. 

## 4. Discussion

In the present study, we showed that high levels of cadmium exposure can induce early menarche. The average menarche age was approximately one year younger in subjects in the heavily polluted area compared with the subjects in the control area.

Previous data showed that the main contributors to dietary cadmium are rice, vegetables, and pork in the Chinese diet [7,8]. For our populations, 80% of dietary cadmium is from rice because the concentrations of cadmium in rice are high, which may be different from other general populations [7]. The dietary pattern of our population is similar to the traditional southern dietary pattern (high intake of rice, vegetables, and pork) [7].

Many factors will affect the timing of pubertal onset, such as nutrition, obesity, and environmental contaminants [9]. Heavy metal exposure is also a factor that may influence changes in the timing of pubertal onset. Previous studies show that lead exposure is associated with later menarche [10,11]. Cadmium exposure results in uterine hyperplasia and early onset of puberty in female rats [1]. However, the effects of cadmium on menarche and menopause in humans have not been fully investigated [12]. The age of menarche is younger than in the past [5]. It has been shown that endocrine disrupting chemicals may play a critical role in this phenomenon [11]. Our data further confirmed that cadmium exposure is associated with the shift in age of menarche. A recent study showed that a higher cadmium body burden (unadjusted urinary cadmium (UCd) > 0.4 μg/L) was less likely to have attained menarche during two years follow-up [13]. The cadmium levels were lower than the exposure in our study. In addition, the study did not show the association between adjusted UCd (shown as μg/g crentinine) and menarche. Moreover, only 12 to 15-year-old girls were included in that study. Further studies are needed. It has been shown that cadmium could be regarded as a potent nonsteroidal estrogen [1]. Cadmium may mimic the effects of estradiol in promoting uterine hyperplasia, development of mammary glands, and earlier menarche [1]. In addition, cadmium can activate membrane-bound estrogen receptors [14] and enhance the activity of estrogen. 

The association between cadmium exposure and age at menopause has not been clarified [5,15]. In the present study, we found that there were no significant differences in the timing of menopause between subjects living in the control area and cadmium polluted areas. The prevalence of delayed menopause was slightly increased in cadmium polluted areas compared with that in the control area. Cadmium may mimic the effects of estradiol in delaying the occurrence of menopause. 

There are several limitations in our study. First, we could not obtain the internal dose of cadmium, including urinary cadmium (UCd) and blood cadmium (BCd), when the subjects were at menarche or menopause. Second, the logistic model did not adjust for some confounders, such as nutritional status or body weight when the subjects were young. Third, the population size was relatively small. Further study with larger sample sizes should be performed. 

In conclusion, our data indicated that cadmium exposure is associated with earlier menarche. Cadmium exposure may slightly delay the age of menopause. Further studies are needed because women are more susceptible to cadmium-induced health effects.
